# The Problem with Diagnosis of Intestinal Schistosomiasis

**DOI:** 10.1016/j.ebiom.2017.10.004

**Published:** 2017-10-03

**Authors:** R. Alan Wilson

**Affiliations:** Centre for Immunology and Infection, Department of Biology, University of York, York YO10 5DD, UK

**Keywords:** Schistosome, Diagnosis

Great strides have been made in the control of intestinal schistosomiasis, caused by the parasitic platyhelminths *Schistosoma japonicum* and *S. mansoni*, primarily via mass chemotherapy programmes with Praziquantel. However, a very significant obstacle to disease eradication remains, namely the sensitivity of diagnostic techniques. The adult worms dwell in the hepatic portal blood vessels, laying eggs in the walls of the large and small bowel. These eggs develop over 5–6 days and then produce secretions that facilitate their passage through the tissues to reach the gut lumen, whence they exit the host to continue the life cycle. Parenthetically, it is the eggs that fail to exit but instead embolise in the liver, which initiate the hallmark pathological sequelae of the disease.

The gold standard for diagnosis of an ongoing schistosome infection has for decades been the detection of eggs in a faecal smear. However, there is abundant evidence that the Kato/Katz smear fails to detect low worm burdens, and it has finally been acknowledged that schistosomiasis is more prevalent than previously thought (Colley et al., 2017). Indeed, in work with laboratory infections of *S. mansoni* in baboons, where surrogate estimates can be directly compared with worm burden determined by perfusion, the limit of detection from nine replicate faecal smears was 40 worms, equating to 16 worm pairs (Wilson et al., 2006). (Similar data are not available for *S. japonicum*.) Protocols to enrich eggs from faeces have resulted in either little improvement in sensitivity (Cringoli et al., 2010), or are too complicated and expensive for routine use (Eberl et al., 2002).

The detection of schistosome-derived products in the blood or urine of patients has provided an alternative approach to diagnostics. In particular two circulating antigens from the worm gut, CAA and CCA, have been developed as indicators of ongoing infection. Initially the tests used specific monoclonal antibodies in a sandwich ELISA to detect the antigens in sera and urine but the technique has been progressively improved and converted to dipstick format for non-invasive, point-of-care testing on urine samples (the POC-CCA cassette). Although this appears to be more sensitive than a faecal smear it still has limitations (Colley et al., 2017). Again, in baboon experiments the sandwich ELISA on serum samples had detection thresholds of 24 and 47 worms for CAA and CCA, respectively (Wilson et al., 2006). Enrichment of CAA in baboon serum using centrifugal concentration devices has taken diagnosis close to the ultimate goal of detecting a single worm pair (Corstjens et al., 2014) but is not practicable in a field setting. It is not presently clear if development the CAA and CCA assays has reached its pinnacle of sensitivity or can be further improved.

In work reported here, Cai and colleagues have taken a different serological approach (Cai et al., 2017). Detection of antibodies to schistosome proteins has long been used as a diagnostic in travellers returning from endemic regions (Coltart et al., 2015). Crude extracts of eggs (or adult worms) are used as the coating antigen in an ELISA test and antibodies to eggs can be detected from about six weeks after exposure to the parasite. The test is very sensitive but has one inherent drawback for use in the diagnosis of people in endemic areas. Antibody titres to schistosome antigen mixtures can remain high for months to years after the infection has been cleared, so the test is unable to distinguish between current and previous infections. This factor is especially important in mass chemotherapy programmes aimed at eradication. Cai et al. report on their attempt to improve the specificity of the antibody ELISA by assessing the diagnostic potential of ten *S. japonicum* surface and excretory-secretory antigens previously reported in the literature. The target proteins were produced as bacterial recombinants and purified for ELISA plate coating. The assay was developed using sera from infected mice and subsequently extended to cohorts of human populations in endemic areas of the Philippines. In addition, the mice were given curative chemotherapy to eliminate the worm population and the decline in circulating antibodies to key targets was followed over several months. Three of the targets performed well in the mouse tests. Two were saposins (SjSAP4 and SjSAP5), lipid binding proteins secreted by the gut epithelium, and the third was an extrinsic loop of a surface-exposed tetraspanin protein (Sj23-LHD). The titres against the two saposins largely did not reduce with time after cure but antibodies to Sj23-LHD had declined to low levels by seven months post-treatment. When human sera were tested, the sensitivity of Sj23-LHD was lower than that of a faecal smear test but the two saposins performed well. The really telling observation was that approximately 50% of people with a negative faecal smear tested positive using the SjSAP4 and SjSAP5 antigens. This result was validated using a sensitive PCR technique to detect schistosome DNA in supposedly negative faecal samples (Cai et al., 2017).

Although these results are preliminary and there is as yet no information about the persistence of human antibody responses to SjSAP4 and SjSAP5, the study does show that the antibody ELISA can be improved as a diagnostic tool by choosing single proteins rather than crude mixtures. Its great strength is its high sensitivity, an essential feature for populations with low worm burdens. However, a note of caution is needed here. Several of the supposed surface and secreted targets investigated by Cai et al., performed poorly as diagnostics (Cai et al., 2017). Upon scrutiny almost all turned out to be internal constituents so new targets need to be chosen with care. Proteomic analyses of adult worm and egg secretions have revealed other potential diagnostic targets (Wilson, 2012). It is plausible that one or more of these might have the optimum characteristics, promoting antibody responses by continuous release from the worm or its eggs while the infection is patent, which then decay rapidly over days to weeks when the worm population is removed by chemotherapy. Such an assay would also be ideal for identifying individuals with only a few worms, below the detection threshold of faecal or circulating antigen tests. With such a specific and sensitive tool, eradication of intestinal schistosomiasis would become a realistic prospect.

## Disclosure

The author declares no conflicts of interest.
